# Profile of HLA-B27-positive enthesitis/spondylitis-related arthritis in Senegal, West Africa

**DOI:** 10.1186/s12969-024-00969-1

**Published:** 2024-02-29

**Authors:** Mounib M. Sabounji, Aïssatou Ndiaye, Saïdou Diallo

**Affiliations:** Department of Rheumatology, Aristide Le Dantec Hospital, Dakar, Senegal

**Keywords:** Enthesitis-related arthritis, Sacroiliitis, Positive HLA-B27, Disease activity, Functional disability, Senegal

## Abstract

**Background:**

Enthesitis/spondylitis-related arthritis (ERA) is a type of juvenile idiopathic arthritis (JIA) frequently associated with HLA-B27. In sub-Saharan Africa, HLA-B27-positive ERA hasn’t been the subject of a specific study.

**Objectives:**

We aimed to describe the clinical features, disease activity, functional disability and treatment of HLA-B27-positive ERA at diagnosis in Senegal and compare the findings to other populations.

**Methods:**

We conducted a retrospective study by reviewing the medical records of patients diagnosed with ERA with an age of symptom onset < 18 years according to the 2019 PRINTO provisional criteria for ERA from January 2012 to December 2022. We collected demographic, clinical, paraclinical and therapeutic data. Disease activity score was assessed by Ankylosing Spondylitis Disease Activity Score (ASDAS) and Bath Ankylosing Spondylitis Disease Activity Index (BASDAI). Functional disability was assessed using Bath Ankylosing Spondylitis Functional Index (BASFI).

**Results:**

A total of 31 patients with HLA-B27-positive ERA were included. Twenty of 31 (64.5%) were males. Twenty-seven (87%) were Fula (ethnicity). The median age at symptom onset and at diagnosis was 12 years and 19 years, respectively. Seven patients had a family history of Spondyloarthritis. Peripheral arthritis and enthesitis were the most common presenting features at disease onset. Peripheral arthritis was present in 29 (93.5%) and located in the lower limbs in 27/29 (93.1%) patients. Heel enthesitis was present in 26 (83.8%) patients. Axial involvement was present in 27 (87%) patients, dominated by low back pain and sacroiliac pain/ buttock pain in 24 (88.8%) and 22 (81.5%) patients, respectively. Seven (22.5%) patients had anterior uveitis. The ESR and CRP were elevated in 65.5% and 57.1% of cases, respectively. On imaging, sacroiliitis was found in 22 patients. The mean BASDAI was 5.5/10 (77.2% of patients had a high active disease; BASDAI ≥ 4/10). The mean ASDAS-ESR/CRP was 3.8. The mean BASFI was 5.4/10 (80% of patients had high functional disability; BASFI ≥ 4/10). Twenty-seven (87%) patients were treated with methotrexate and non-steroidal anti-inflammatory drugs. After 6 months of treatment, mean BASDAI was 3/10 and mean BASFI was 2.5/10.

**Conclusion:**

In our study, HLA-B27-positive ERA was found in our Senegalese cohort mainly in adolescents of the Fula ethnic group. 22 (70.9%) patients developed ankylosing spondylitis at adulthood. The disease was very active at the time of diagnosis with significant functional disability. Treatment was mainly based on methotrexate and NAISDs.

## Introduction

Enthesitis/spondylitis-related arthritis (ERA) is a type of juvenile idiopathic arthritis frequently associated with HLA-B27 [1, 2]. It’s a chronic inflammatory disease characterized by enthesitis, peripheral arthritis predominantly in the lower limbs and axial arthritis (sacroiliitis and spondylitis) which can progress to ankylosing spondylitis at an advanced stage, occurring before 18 years [1, 2]. It is distinguished by male predominance, a strong association with HLA-B27 and the development of anterior uveitis [3]. ERA represents a continuum with adult spondyloarthritis (SpA) and children with HLA-B27-positive ERA are associated with a prolonged disease course and higher incidence of anterior uveitis [4, 5].

 In sub-Saharan Africa, spondyloarthritis is generally uncommon because the majority of the population is considered to be negative HLA-B27 [6]. However, an exception has been found in the Fula (Peul) ethnic group of Gambia (West Africa), where the overall prevalence of the HLA-B27 antigen is 6-7.8% [7, 8]. This ethnic group is also present in Senegal and other West African countries.

 To our knowledge, HLA-B27-positive ERA has never been studied specifically in our region. Thus, this study aims to describe demographic characteristics, clinical features, disease activity, functional disability and treatment of HLA-B27-positive ERA at a tertiary hospital in Senegal (West Africa).

## Patients and methods

This retrospective cohort study was carried out at the rheumatology department of Aristide Le Dantec Hospital in Dakar, from January 2012 to December 2022. All patients who fulfilled the 2019 PRINTO preliminary criteria of enthesitis/spondylitis-related JIA: arthritis for ≥ 6 weeks, enthesitis, inflammatory back pain > 3 months, sacroiliac joint tenderness, anterior uveitis, history of a SpA in relative, positive HLA-B27, with age of onset under 18 years were enrolled in the study [2]. Exclusion criteria were systemic JIA, juvenile psoriasis arthritis, RF-positive JIA (childhood-onset rheumatoid arthritis), acute rheumatic fever, and post-infectious arthritis. The following data were collected:

- Age at symptom onset and at diagnosis (presentation), duration of symptoms (defined as the delay between symptom onset and presentation at rheumatology department), sex (female, male) and ethnicity.
- Clinical features: arthritis (defined as swelling within a joint or limitation in the range of joint movement with joint pain or tenderness), enthesitis (defined as tenderness on palpation of entheseal sites; Heel enthesitis is defined as past or present spontaneous pain or tenderness at the examination of the site of the insertion of the Achilles tendon or plantar fascia at the calcaneus), Sacroiliac tenderness (defined as tenderness on palpation of the SI joints and/or pain on SI manoeuvres) and Uveitis (defined as past or present uveitis anterior, confirmed by an ophthalmologist).
- Laboratory investigations: positive HLA-B27, erythrocyte sedimentation rate (ESR, first hour; raised if > 20 mm/hour), C-reactive protein (CRP; positive if > 6 mg/l).
- Imaging investigations: radiographs and/or computed tomography of sacroiliac Joints. Sacroiliitis is defined as the presence of erosions and/or sclerosis in accordance with the modified New York criteria (at least grade 2 bilateral or grade 3 unilateral). In our context MRI was unavailable.
- Disease activity was assessed by: Ankylosing Spondylitis Disease Activity Score (ASDAS by ESR or CRP, ASDAS ≥ 2.1 defined as active disease) [9], and Bath Ankylosing Spondylitis Disease Activity Index (BASDAI ≥ 4/10 defined as active disease) [10, 11].
- Physical function at diagnosis was assessed using the Bath Ankylosing Spondylitis Functional Index (BASFI ≥ 4/10 is defined as high disability) [12, 13].
- Treatment options were noted: conventional synthetic disease-modifying anti-rheumatic drugs (csDMARDs): methotrexate, sulfasalazine. Corticoids (intra-articular injections). Non-steroidal anti-inflammatory drugs (NSAIDs).
- Follow-up: after initiation of treatment, we follow up patients for six months. Disease activity and physical function were assessed after this period of therapy using the BASDAI and BASFI.

Given the nature of this study, informed consent for participation was not required for retrospective data. Confidentiality was ensured for all participants.

### Data analysis

Statistical analysis of data was done using Statistical Package for Social Sciences (SPSS) version 21.0 for Windows. Descriptive analysis was done and statistics were presented as numbers and percentages for categorical data and mean and standard deviation (SD) for continuous data. The Pearson correlation coefficient was used. *P*-values < 0.05 was considered statistically significant.

## Results

### Demographic and clinical features

A total of 40 patients were enrolled, all met the 2019 PRINTO criteria for ERA. Among them, 31 (77.5%) had positive HLA-B27. Descriptive data for all 31 patients are presented in Table 1. The male-to-female ratio was 1.8 (20 M:11 F). The mean age at symptom onset and at the time of diagnosis was 11.8 ± 2.9 (median: 12 years) and 19.8 ± 8.4 years (median: 19 years), respectively. Among 31 patients, 27 (87%) were Fula (ethnicity). The median duration of symptoms was 6.0 years.

**Table 1:** Demographic and clinical characteristics of ERA patients with HLA-B27-positive at diagnosis

Variable	Number (%)	Median (± SD)	Range
**Sex**			
Male	20 (64.5)		
Female	11 (35.5)		
**Age (years)**			
Age at symptom onset		12 (± 2.9)	7–16
Age at diagnosis (presentation)		19 (± 8.1)	7–45
**Ethnic Groups**			
Fula ethnic group	27 (87)		
Wolof ethnic group	2 (6.4)		
Other ethnic group	2 (6.4)		
**Peripheral joint involvement**	29		
Arthritis in the lower limbs	27 (93.1)		
Knee joint involvement	23 (85.1)		
Hip joint involvement	13 (48.1)		
Ankle joint	10 (37)		
Foot joint	8 (29.6)		
**Axial joint involvement**	27		
Sacroiliac pain / Buttock pain	22 (81.5)		
Low back pain	24 (88.8)		
Cervical pain	5 (18.5)		
Dorsal pain	10 (37)		
**Enthesitis involvement**	26		
Heel enthesitis	26		
Anterior chest wall	8		
**Extra-articular involvement**	7		
Anterior uveitis	7 (22.5)		
**Familial history of SpA**	7 (22.5)		
**BASFI score**		5.8 ± 1.5	1.8–8.8

**Fig. 1:**
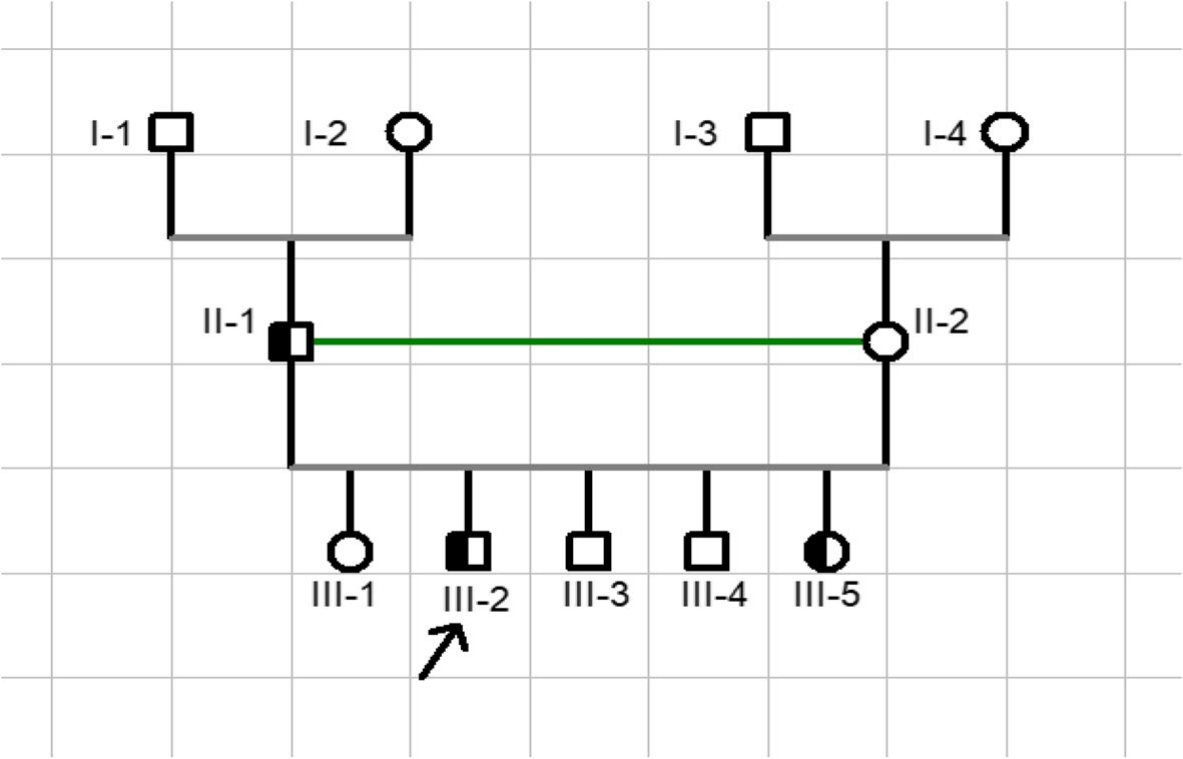
Familial Case of HLA-B27-positive SpA in Fula ethnic group, consanguine marriage between II-1 and II-2; index case (III-2) 29 years old male with HLA-B27 positive axial and peripheral SpA, his father (II-1) with axial SpA, his sister (III-5) 17 years old female with HLA-B27-positive ERA (circles and squares indicate females and males respectively)

 The inaugural symptoms reported by our patients at anamnesis were: peripheral joint involvement in 25 (80.6%) patients, entheseal involvement especially posterior and plantar talalgia in 19 (61.3%) patients, axial joint involvement in 16 (51.6%) patients. Four (12.9%) patients had anterior uveitis as the initial manifestation of ERA. Seven patients had a family history of SpA. Figure 1, illustrates a familial case of HLA-B27 positive spondyloarthritis in this study (notion of consanguineous marriage in a Fula ethnic group).

At the time of presentation, peripheral involvement was present in 29 (93.5%) patients. Peripheral arthritis was located in the lower limbs in 27 (93.1%) patients (Table 1). The most commonly affected joint was the knee (84%), followed by ankle (44%), hips (32%), and foot joints (24%). Axial involvement was present in 27 (87%) patients, dominated by low back pain in 24 (88.8%) patients followed by sacroiliac pain/ buttock pain in 22 (88.5%), dorsal pain in 10 of 27 and cervical pain in 5 of 27 patients. Two patients had axial arthritis without peripheral involvement. Entheseal involvement was dominated by heel enthesitis in 26 cases, followed by the anterior chest wall in 8 patients. Among extra-articular manifestations, anterior uveitis was present in 7 (22.5%) patients.

**Fig. 2:**
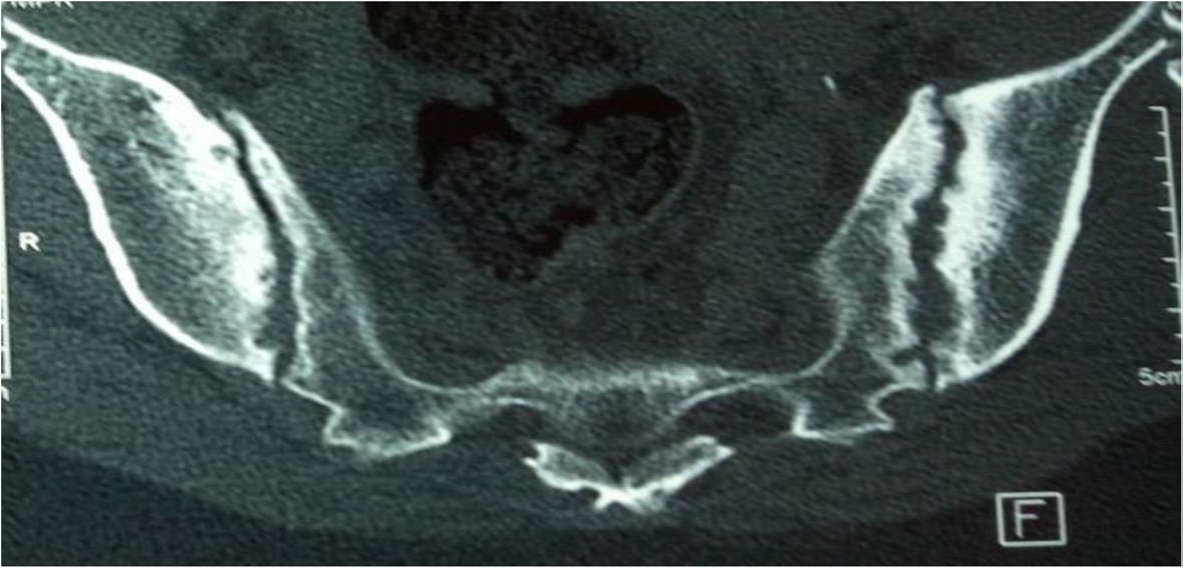
Bilateral sacroiliitis with erosions and subchondral sclerosis (Grade 3) in a 30-year-old female with HLA-B27-positive ERA (14 years’ disease duration)

Among 27 patients with axial involvement, 25 underwent radiography and/or computed tomography of the sacroiliac joints. Sacroiliitis was found in 22 (88%) patients. These patients with radiographic sacroiliitis represented the advanced form known as ankylosing spondylitis. Figure 2 shows grade 3 bilateral sacroiliitis on CT in a female with HLA-B27 positive ERA.

In terms of functional disability, 20 patients completed the BASFI questionnaire. The median BASFI score was 5.8/10 (mean 5.4/10) at diagnosis and 16 (80%) patients had a high functional disability (BASFI > 4/10).

### Laboratory features and disease activity at diagnosis

Inflammatory biomarkers were elevated in almost one out of two patients. The ESR was accelerated in 19 (65.5%) patients, with an average of 36.04 mm/h. While CRP was elevated in 16 (57.1%) patients, with an average of 27.8 mg/l (Table 2). In terms of disease activity, 22 patients completed the BASDAI questionnaire. the mean BASDAI was 5.5 ± 1.9 and 77.2% (17/22) of patients had a high active disease (BASDAI > 4/10). The mean ASDAS was 3.8 ± 0.9. Functional disability (BASFI) was correlated with BASDAI (*r* = 0.72, *P* < 0.01) and ASDAS (*r* = 0.6, *P* < 0.05).

**Table 2:** Laboratory characteristics and disease activity of HLA-B27 positive ERA patients at diagnosis

Laboratory abnormalities	Total number of cases tested	Number (%)
Raised ESR (ESR ≥ 20 mm/h)	29	19 (65.5)
Elevated CRP (CRP > 6 mg/l)	28	16 (57.1)
**Laboratory parameters**	**Mean (± SD)**	**Range**
ESR (mm/h)	36.04 (± 23)	5–90
CRP (mg/l)	27.8 (± 44)	1-192
**Disease activity score**	**Mean (± SD)**	**Range**
ASDAS-ESR/CRP	3.8 (± 0.9)	2.65–6.06
BASDAI	5.5 (± 1.9)	1.2-9

### Therapeutic regime and follow-up

A wild majority of patients (27 of 31) were treated with methotrexate and NSAIDs, while four patients had a combination of sulfasalazine and NSAIDs. Approximately, a third of patients had intra-articular steroid injections. After six months of treatment, the average BASDAI was 3.2/10 with a median of 2.95 ± 0.96. The mean BASFI was 2.49/10.

## Discussion

To our knowledge, this study is the first in sub-Saharan Africa to specifically describe the profile of HLA-B27-positive ERA. Previous studies have described ERA in the context of juvenile idiopathic arthritis using ILAR criteria without HLA-B27 screening [14, 15].

In Senegal, three ethnic groups predominate Wolof (43%), Fula (Peul) (24%) and Serer (15%) representing a total of 82% of the Senegalese population. Our study shows that HLA-B27-positive ERA mainly affects the Fula ethnic group (87%), even though this ethnic group represents only 24% of the population. This result was linked to the high prevalence of HLA-B27 (6-7.8%) in this ethnic group compared with the rest of the sub-Saharan African population, which is generally less than 1% [6–8]. Another factor is the high frequency of endogamy (consanguineous marriage) within this ethnic group, as illustrated in Fig. 1.

In this study, there was a male predominance (64.5%) which was generally consistent with the findings of other HLA-B27-positive ERA cohort studies (Table 3). The mean age of onset in our patients was approximately 12 years, which was similar to that reported in the literature [5, 16–19]. However, an earlier median age has been reported in a Turkish study (10 years) [20].

**Table 3:** Comparative Data for HLA-B27-positive ERA

Study	Singapore [16]	India [5]	USA, Italy [17]	Poland [18]	Bulgaria [19]	Turkey [20]	Senegal
Number	120	109	69	44	55	36	31
Male, n (%)	105 (87.5)	NA	50 (72.5)	31 (70.4)	44 (80)	29 (80.6)	20 (64.5)
Age at onset	12.1	12.7	12.3	12.5	11.6	10.0	11.8
Disease duration (year)	0,25	3.0	NA	NA	NA	2.0	6.0
Sacroiliitis, n (%)	68 (56.7)	56 (51.4)	15^a^(83.3)	NA	34 (61.8)	19 (52.7)	22 (88)
Peripheral Arthritis, n (%)	107 (89.2)	34 (63)	50^b^(72.5)	NA	NA	23 (63.8)	29 (93.5)
Enthesitis, n (%)	51 (42.5)	69 (63.3)	NA	NA	24 (43.6)	16 (44.4)	26 (83.8)
Uveitis, %	4.2	14.7	5.8	NA	NA	22.2	22.5
ESR mm/1st H	34	50	NA	NA	NA	30	36

*NA N*ot available

^a^denominators varied due to missing data

^b^arthritis in a male over 6 years of age

The long duration of symptoms (median: 6.0 years) is linked to several factors which constitute barriers to early diagnosis and treatment in our context. We can suggest three possible explanations for that. Firstly, some patients initially favour the use of traditional medicines (phytotherapy), before consulting in the hospital at a later stage. Secondly, the unawareness of this disease in primary health care facilities, which treat it symptomatically (analgesics) without early referral to rheumatology department. Thirdly, the scarcity of rheumatology specialists in Senegal. This delay highlights the need to raise awareness of this diagnosis and facilitate earlier recognition and referral to rheumatologist.

The median age of our patients (19 years) at diagnosis reflects the progressive nature of the disease, starting in childhood and continuing into adulthood. This confirms that ERA represent a continuum with adult spondyloarthritis [4].

Remarkably, approximately 22% of patients reported a family history of SpA, supporting a major role for genetic factors. This result was in agreement with the Indian study [5].

In our cohort, peripheral arthritis and enthesitis were the most common presenting features at disease onset. This finding is related to the natural course of the disease. Spondyloarthritis usually begins in children with peripheral arthritis predominantly involving the lower limbs and/or with peripheral enthesitis rather than axial arthritis [1, 21, 22].

Enthesitis is a hallmark feature of ERA [23, 24] and reported at the time of diagnosis in 44.4–70% of patients with HLA-B27 positive ERA [5, 20, 25]. In the current study, we found a relatively high rate (83.8%) of enthesitis compared to previous studies.

The majority of our patients with peripheral arthritis had predominantly knee and ankle joint involvement. These findings were in accordance with previously published HLA-B27 positive ERA series [17, 19, 20]. The frequency of sacroiliitis at disease presentation in our study (88%) was higher than in reported studies (51.3 to 83.3%). This frequency is linked to the long duration of the disease (6 years) in our patients in contrast with previous studies.

Uveitis was the most common extra-articular manifestation of HLA-B27-positive ERA [16, 20]. In this current study, the frequency of uveitis was (22.5%). This result was in agreement with the Turkish study (22.2%). However, lower prevalence was reported in Indian (14.7%) and American (13%) studies [5, 26].

Concerning the assessment of enthesitis/spondylitis related arthritis, we opted to use the BASDAI, ASDAS and BASFI to assess patients with ERA. Given that the majority of patients presented the advanced form of ERA known as ankylosing spondylitis or radiographic axial SpA. Indeed, Batthish et al. demonstrated that both the BASDAI and BASFI showed excellent intra-rater reliability in a cohort of ERA patients [27]. Our study demonstrates that there is a positive correlation between disease activity (ASDAS, BASDAI) and BASFI. This indicates that active inflammation has an impact on physical function.

Regarding drug therapy, although biological agents (TNFα inhibitors) are included in the WHO (World Health Organisation) essential medicines list for paediatric rheumatology [28], they are unavailable in our context. The therapeutic options currently available are NSAIDs, glucocorticoids and conventional synthetic disease-modifying anti-rheumatic drugs (methotrexate, sulfasalazine). In Senegal, we use a combination of methotrexate/sulfasalazine and NSAIDs associated with glucocorticoids (intra-articular injections).

Several limitations of our study should be noted. This cohort was retrospective and other extra-articular manifestations such as cardio-vascular involvement was not studied. Magnetic resonance imaging was not used in our study due to a lack of resources. The disease activity at diagnosis was probably under-evaluated because some patients initially received analgesic treatment in primary health before referral.

 Despite the study’s limitations, this research is highly relevant to future studies on juvenile spondyloarthritis in sub-Saharan African populations, which are generally under-represented in medical research.

## Conclusion

In our study, HLA-positive ERA was found in our Senegalese cohort mainly in adolescents of the Fula ethnic group. It is characterized by peripheral arthritis, sacroiliitis and heel enthesitis. The disease was active at the time of diagnosis with significant functional disability. Twenty-two (70.9%) patients developed ankylosing spondylitis at adulthood. The treatment was mainly based on NSAIDs and methotrexate. However, further prospective studies are needed, including research into genetic factors, to obtain reliable clinical phenotypes of this disease, particularly in sub-Saharan Africa.

## Data Availability

The datasets from this study are available on request from the corresponding author.

## Abbreviations

ERA: Enthesitis/spondylitis related arthritis
SpA: Spondyloarthritis
JIA: Juvenile idiopathic arthritis
PRINTO: Paediatric Rheumatology International Trials Organization
csDMARDs: Conventional synthetic disease-modifying anti-rheumatic drugs
